# Chirurgisches Nahtmaterial – Grundlagen

**DOI:** 10.1007/s00064-023-00812-y

**Published:** 2023-08-21

**Authors:** Klaus Dresing, Theddy Slongo

**Affiliations:** 1 Klinik für Unfallchirurgie, Orthopädie und Plastische Chirurgie, Universitätsmedizin Göttingen, Robert-Koch-Str. 40, 37075 Göttingen, Deutschland; 2 Dept. of Paediatric Surgery, University Children’s Hospital, 3010 Bern, Schweiz

**Keywords:** Eigenschaften, Knotentechniken, Techniken der Fadenentfernung, Fadenstärken, Fadenzug, Properties, Knotting techniques, Suture removal techniques, Suture strengths, Suture removal

## Abstract

Am Ende der chirurgischen Therapie wird der Zugang mit Nahtmaterial geschlossen. Chirurgische Nähte werden also zur Adaptation von Wundrändern und Gewebe verwendet. Aufgabe des Nahtmaterials ist, die Gewebe bis zur Heilung zusammenzuhalten. Für Patienten ist eine kosmetisch gute Naht häufig das Zeichen einer guten Chirurgie. Für verschiedene Gewebe und -schichten werden unterschiedliche Anforderungen an das Nahtmaterial gestellt. Es werden die verschiedenen Arten von monofil über polyfil, geflochten, von resorbierbar bis nichtresorbierbar vorgestellt. Die Eingruppierung der Fadenstärken wird verglichen. Die Einsatzgebiete verschiedener Fadenstärken und die Dauer bis zum Fadenzug in den verschiedenen Körperregionen werden vorgestellt. Detailliert werden die Einhand- und Zweihandtechnik der chirurgischen Knoten erläutert. Die Technik der Nahtmaterialentfernung wird präsentiert. Im Online-Material können die Nahtmaterialien verschiedener Zusammensetzung und Hersteller gegenübergestellt werden.

**Zusatzmaterial online:**

Zusätzliche Informationen sind in der Online-Version dieses Artikels (10.1007/s00064-023-00812-y) enthalten.

## Abstract (en)

At the end of surgical therapy, the access is closed with sutures. Surgical sutures are thus used to adapt wound edges and tissues. The task of the suture material is to hold the tissues together until healing. For patients, a cosmetically good suture is often the sign of good surgery. Different tissues and layers have different requirements regarding the suture material. The different types from monofil to polyfil, braided, from absorbable to nonabsorbable are presented. The classification of suture strengths is compared. The areas of application of different strengths and the duration until removal of the sutures for the different body regions are presented. The one-hand and two-hand techniques of surgical knots are explained in detail. The technique of suture removal is presented. In the online material, the suture materials of different composition and manufacturers can be compared.

## Lernziele

Nach der Lektüre dieses Beitrags ...

- kennen Sie die verschiedenen Arten und Formen von Nahtmaterialien,
- können Sie resorbierbare von nichtresorbierbaren Materialien unterscheiden,
- sind Ihnen die Eigenschaften, Vor- und Nachteile von monofilem und polyfilem Nahtmaterial bekannt,
- sind Sie in der Lage, die Techniken der chirurgischen Knoten zu beschreiben und nach Übung zu praktizieren,
- ist Ihnen die Technik des Fadenzugs bekannt, und Sie können diese durchführen.

## Einleitung

Chirurgische Nähte werden zur Adaptation von Wundrändern und Gewebe verwendet, ohne dabei übermäßige, schädigende Spannung im Sinne von Ischämie zu verursachen [1]. Das **Nahtmaterial**

Nahtmaterial

muss dabei in der Lage sein, das Gewebe bis zur vollständigen Heilung zusammenhalten zu können. Bei **resorbierbaren Nähten**

resorbierbaren Nähten

darf die Resorption erst nach der Heilung einsetzen. Im Weiteren soll das Nahtmaterial für den Chirurgen sicher und einfach anwendbar und an die zu nähenden Strukturen angepasst sein. Das Nahtmaterial selbst sollte dabei möglichst geringe Interaktion mit dem Gewebe hervorrufen.

Oft setzt der Patient die sichtbare Wunde (Hautnaht) respektive die später sichtbare Narbe mit der Qualität oder Erfolg der Operation gleich. Wir wissen jedoch, und dies sollte dem Patienten vor jeder Operation schon gesagt werden, dass nicht jeder Mensch eine identische **Narbenbildung**

Narbenbildung

hat. Kinder und Jugendliche neigen viel stärker zur hypertrophen Narbenbildung (bei uns fälschlicherweise als Keloid bezeichnet), wogegen ältere Menschen aufgrund der Atrophie des Gewebes kosmetisch sehr ansprechende Narben aufweisen können. Das innere funktionelle Ergebnis wie auch die äußere sichtbare Narbe können durch nichtadäquates Nahtmaterial und Nahttechnik, aber auch durch eine inadäquate Inzision (Hautlinien) sehr stark leiden. Im Weiteren hängt das Resultat der Narbe nicht zuletzt auch von der **Wundspannung**

Wundspannung

bei Schwellung oder durch übermäßige Hautexzision (z. B. Narbenkorrektur) ab [2, 3].

Prinzipiell können folgende Faktoren die Qualität (Wahrnehmung) einer Naht beeinflussen:

- Entstehungsart der Wunde (elektiv oder traumatisch),
- Lokalisation der Wunde,
- Hautdicke/Hautbeschaffenheit,
- Spannung der Weichteile,
- kosmetisches Ziel/Erwartungshaltung (dies ist eine Wahrnehmung),
- Infekte,
- Anordnung der Einstichstellen:
  - Distanz zum Wundrand,
  - konstanter Abstand der einzelnen Einstichstellen,
- Alter des Patienten.

### Nahtmaterial

Die Wahl des Nahtmaterials hängt einerseits von der Körperregion, der zu erwartenden Spannung des Gewebes bzw. der Wunde ab, andererseits aber auch vom Zustand des Gewebes selbst. Die verschiedenen Nahtmaterialien lassen sich nach Zugfestigkeit, Knotenfestigkeit, Handhabung und Gewebereaktion sowie nach Resorbierbarkeit einteilen. Welchen Ansprüchen das **ideale Nahtmaterial**

ideale Nahtmaterial

in Bezug auf den zu erfüllenden Zweck genügen muss, hat der Chirurg zu definieren und danach zu wählen. Wunsch des Operateurs ist die Kombination von hoher Zugfestigkeit, einfacher Handhabung, geringer bis keiner Gewebereaktivität und minimalem Infektionsrisiko. Das Material soll gut im Gewebe sichtbar sein, und es soll aus nichtkapillarem, nichtallergenem und nichtkarzinogenem Material bestehen [4].

Es werden hauptsächlich 2 Gruppen unterschieden: resorbierbares und nichtresorbierbares Nahtmaterial. **Resorbierbare Fäden**

Resorbierbare Fäden

verlieren ihre Zugfestigkeit meist in weniger als 60 Tagen. Diese werden in der Regel nicht entfernt.

**Nichtresorbierbare Fäden**

Nichtresorbierbare Fäden

behalten den Hauptteil der Zugfestigkeit über 2 Monate hinaus. Diese auch für Hautnähte gebräuchlichen Fäden werden entfernt.

Auf Wundverschluss mit Klammersystemen wird in dieser Arbeit nicht eingegangen.

### Wundheilung

Es wird die primäre von der sekundären Wundheilung unterschieden. In diesem Beitrag soll es weitgehend um chirurgische Wunden nach operativem Management also **primäre Wundheilung**

primäre Wundheilung

gehen.

Die ersten 3 Tage nach Haut- und Weichteilverschluss werden als exsudative oder **inflammatorische Phase**

inflammatorische Phase

charakterisiert. Es folgt die **proliferative Phase**

proliferative Phase

an Tag 4 bis 7 gefolgt von der **reparativen Phase**

reparativen Phase

ab Tag 8 bis zu Monaten. Auf die Darstellung der Details der pathophysiologischen Abläufe wird hier verzichtet. Das Nahtmaterial ist in der ersten Phase für den Zusammenhalt der Wunde verantwortlich.

Nahtmaterialien können im Gewebe **Fremdkörperreaktionen**

Fremdkörperreaktionen

auslösen. Daher sollten bei der Aufklärung zur Operation die Patienten nach bisherigen Reaktionen auf Nahtmaterial gefragt werden.

#### Merke

Es empfiehlt sich, möglichst den dünnsten erforderlichen Faden zu verwenden, um Fremdkörperreaktionen so gering wie möglich zu halten.

## Anforderung an chirurgisches Nahtmaterial

Bei der Beurteilung der Eigenschaften von Nahtmaterial sind verschiedene Parameter zu beachten: physikalische Parameter, Flüssigkeitsaufnahme und Kapillarität, Kaliber oder Durchmesser, Zugfestigkeit, Torsion, Absorptionsfähigkeit, Elastizität, Plastizität, Gedächtnis, Reibungskoeffizient und Knotensicherheit. Die optimalen Bereiche für jede dieser Eigenschaften sind für die meisten Nahtmaterialien und Indikationen noch nicht definiert [4].

Das Nahtmaterial muss auch eine **hohe Biegsamkeit**

hohe Biegsamkeit

(Geschmeidigkeit) und Flexibilität aufweisen, damit es sich beim Nähen besser handhaben lässt. Darüber hinaus sind eine einfache Knotenplatzierung, eine hohe Knotensicherheit, aber auch Reizfreiheit und Schutz vor Infektionen weitere wichtige und zu fordernde Eigenschaften. [2, 3].

Die **verwendeten Materialien**

verwendeten Materialien

unterscheiden sich hinsichtlich:

- Reißfestigkeit/Zugfestigkeit (Zugspannung im Augenblick des Reißens des Nahtmaterials),
- Elastizität (Fähigkeit, nach Dehnung wieder in die Ursprungslänge zurück zu gelangen),
- Plastizität (Eigenschaft, nach Dehnung die neue Länge beizubehalten [3]),
- Memory-Effekt (nach Formveränderung wieder in den ursprünglichen Zustand zurückzukehren [3]),
- bestimmter Durchmesser (Kaliber),
- Festigkeit der Knoten-Oberfläche,
- Aufnahme von Flüssigkeit ins Material (Fähigkeit des Nahtmaterials, Flüssigkeit aufzusaugen, zu absorbieren [5]),
- Kapillarität (Fähigkeit des Nahtmaterials, Flüssigkeit aufzunehmen und im Material weiterzuleiten [5]),
- Eigenschaften in der Handhabung,
- Biegsamkeit (lässt sich leicht biegen) (Geschmeidigkeit),
- Reibungskoeffizient (leichtes Gleiten) – zur Verhinderung von Gewebewiderstand, Verrutschen von Knoten und zur Erleichterung des Knüpfens von Knoten,
- Eigenschaft der Gewebereaktion,
- nicht allergen,
- nicht krebserregend,
- (minimale) Gewebereaktionen,
- physikalische Eigenschaften,
- Reibungskoeffizient,
- Monofilament oder Multifilament,
- Nahtmaterial mit Widerhaken,
- Kapillarität,
- Absorptionsfähigkeit.

### Reißfestigkeit.

Reißfestigkeit (Zugfestigkeit) ist der Kraftaufwand, um einen linear gestreckten Faden zu zerreißen [5].

### Knoten.

Mit **Knotensitz**

Knotensitz

wird das sichere Halten des Knotens auf dem Faden nach Einbringung ins Gewebe bezeichnet. Der Knoten soll im Endzustand nicht auf dem Faden rutschen.

**Knotenreißkraft**

Knotenreißkraft

ist der Kraftaufwand, der benötigt wird, einen geknoteten Faden zu zerreißen. Es ergeben sich definierte Prüfwerte. Die **Knotenbruchfestigkeit**

Knotenbruchfestigkeit

sagt aus, bei welcher Kraft der Faden im Knoten reißt. Im Knoten hat der Faden den schwächsten Punkt der Naht.

### Torsion.

Sie wird durch die Anzahl der Verdrehungen im Faden dargestellt. Diese steht in umgekehrtem Verhältnis zur Zugfestigkeit der Naht. Eine Vergrößerung des Durchmessers einer Naht führt zu einer Erhöhung der **Längskraft**

Längskraft,

die erforderlich ist, um die Naht zu zerreißen; eine Verdoppelung des Durchmessers der Naht erfordert eine Vervierfachung des zum Zerreißen der Naht erforderlichen Gewichts [6].

### Dochtwirkung.

Dochtwirkung fußt auf der Kapillarität des Fadens. Nur die **monofile Fadenkonstruktion**

monofile Fadenkonstruktion

gibt zuverlässigen Schutz vor Bakterientransport oder -migration [7]. Die multifilen und pseudomonofilen Fadenkonstruktionen fördern ausnahmslos die Übertragung von Bakterien, wenn auch in unterschiedlichem Maße [7]. Das Eindringen von Flüssigkeiten und Bakterien ist abhängig von den Absorptionseigenschaften, der Beschichtung und dem Vorhandensein eines offenen Fadenendes [7]. Auch die Bindung von Bakterien an das Nahtmaterial ist abhängig von dem Fadentyp, von Material und Oberfläche. Geflochtenes, also polyfiles resorbierbares Nahtmaterial sollte nicht bei infektiösem Gewebe verwendet werden [8].

### Quellung.

Quellung ist das Aufsaugen von Flüssigkeit (**Wundsekret**

Wundsekret)

vom Nahtmaterial.

### Gewebedurchzug.

Hiermit ist das Gleiten des Nahtmaterials durch das Gewebe charakterisiert.

### Sterilität.

Sterilität wird durch **Sterilisationsverfahren**

Sterilisationsverfahren

erzielt, Chromierung z. B. bei Katgut.

### Elongation.

Elongation ist die Ausdehnung des Nahtmaterials. Diese kann temporär oder auch dauerhaft auftreten.

### Resorption.

Die **Resorptionszeit**

Resorptionszeit

gibt den Zeitpunkt an, bis zu dem das gesamte Material abgebaut wird. Die Halbwertszeit gibt den Zeitpunkt an, bis zu dem noch 50 % der Ausgangsreißkraft vorliegt (s. oben).

## Auswahl des Nahtmaterials

Die Unterscheidung zwischen traumatischem und atraumatischem Nahtmaterial bezieht sich auf die **Nadel-Faden-Verbindung**

Nadel-Faden-Verbindung

[9]. **Traumatische Fäden**

Traumatische Fäden

werden in das Öhr der Nadel eingefädelt, während **atraumatisches Nahtmaterial**

atraumatisches Nahtmaterial

in der Nadel mittels Quetschung, Verklebung oder Laserung quasi ohne Durchmesseränderung fixiert ist [9].

Bei der Auswahl des Nahtmaterials gilt der Grundsatz, mit einem Minimum an unerwünschten Gewebereaktionen und infektiösem Potenzial eine ausreichende **Festigkeit der Wunde**

**Figure 1:**
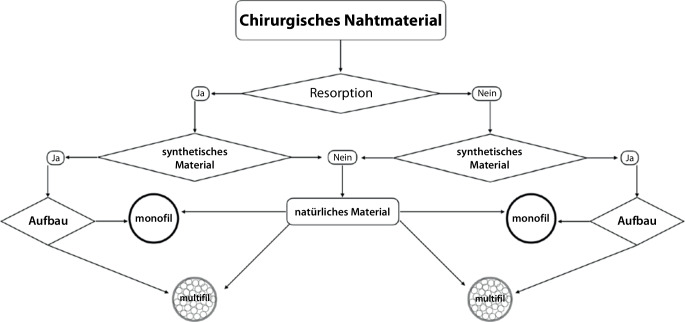

Festigkeit der Wunde

für die notwendige Dauer der Wundheilung zu erreichen. Stärkeres oder zugfesteres Nahtmaterial ist jedoch nicht immer besser, da das Fadenkaliber erhöht werden muss und es zu einer unbeabsichtigten Strangulierung des Gewebes kommen kann, was einerseits die Durchblutung gefährdet, möglicherweise auch die Entzündungsreaktivität erhöht [10]. Die einzelnen Gewebe, Schichten und Indikationen erfordern Konditionen von der Sehnenreparatur bis zur feinen kosmetischen Naht im Gesicht (Abb. 1).

### Aufbau des Nahtmaterials

**Figure 2:**
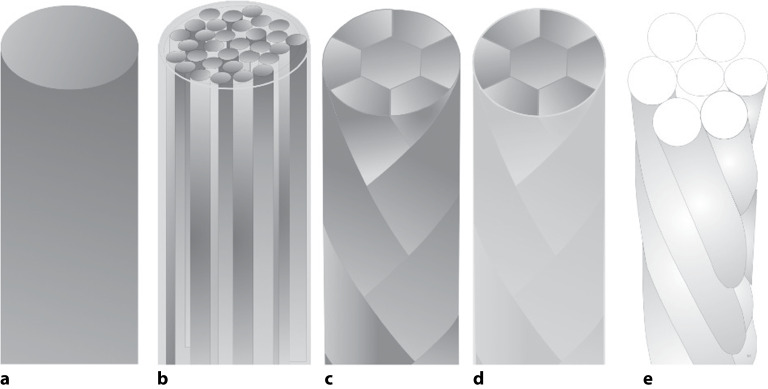

Nahtmaterial wird in verschiedenen Konfigurationen angeboten. Beim Aufbau von Nahtmaterial wird unterschieden zwischen monofilem, poly- oder multifilem Aufbau. Dabei können auch zusätzlich Beschichtungen oder Ummantelungen aufgebracht sein (s. Tabelle A2; Abb. 2).

#### Monofil

Monofil bezeichnet man ein **einzelsträngiges Filamentnahtmaterial**

einzelsträngiges Filamentnahtmaterial

(z. B. Polyamid 6 oder Nylon). Diese Fäden werden mit dem Verfahren der Extrudierung hergestellt. Kapillarität innerhalb des Nahtmaterials tritt nicht auf, die Oberfläche ist glatt, wodurch das Infektionsrisiko geringer ist. Die Gewebepenetration bzw. der Gewebedurchzug wird durch die glatte Oberfläche erleichtert. Aufgrund verminderter Reibung aufgrund der glatten Oberfläche ist dagegen die Haltekraft der Knoten vermindert, sodass normalerweise mehr Knoten als bei multifilem Material gelegt werden müssen. Das Fadenmaterial kann bei der Handhabung etwas rigide erscheinen.

#### Polyfil/multifil

Bei den multiplen Fäden werden mehrere Einzelfäden (Filamente) verdreht, geflochten oder verzwirnt. Auf dem Boden der **vermehrten Rauigkeit**

vermehrten Rauigkeit

und damit höheren Reibung halten Knoten dieses Materials besser.

##### Merke

Bei geflochtenen Fäden halten die Knoten aufgrund der Oberflächenrauigkeit und damit höheren Reibehaftung besser.

Die rauere Oberflächenbeschaffenheit führt beim Durchzug des Fadens durch das Gewebe zu einer Sägewirkung des Fadens. Werden diese Fäden in steilem Winkel durch das Gewebe gezogen, ist die Sägewirkung des Nahtmaterials deutlich geringer als im flachen Winkel.

##### Merke

Bei geflochtenem Nahtmaterial wird mit einem steilen Eintrittswinkel zur Oberfläche die Sägewirkung des Fadens im Gewebe verringert.

Bei diesen Fäden kann auch auf dem Boden der Kapillarität eine **Dochtwirkung**

Dochtwirkung

entstehen, die den Transport von Keimen in die Wunde entlang des Fadens begünstigen kann. Je glatter die Oberflache ist – z. B. Vergleich monofiler Polyesterfaden und polyfiler Polyesterfaden –, desto geringer ist die Anzahl an Bindegewebe- und Fremdkörperriesenzellen, die sich in und um den Faden nach Implantation ansammeln [11]. Hierbei ist die Dochtwirkung bei geflochtenen Fäden geringer als bei gezwirnten, da die Einzelfilamente beim Flechten fast quer zur Längsachse liegen. Insgesamt ist bei geflochtenem Nahtmaterial die Oberfläche größer, und Todräume innerhalb des Fadens sind vermehrt, sodass dadurch die potenzielle Bakterienansiedlung gefördert wird [12].

Das Material wirkt beim Knoten geschmeidig, der Knotensitz aufgrund der Reibung ist gut, die Reißkraft ist hoch.

Durch Beschichtung multipler Fäden wird die Reibekraft der Oberfläche reduziert und ein besserer Durchzug erzielt.

#### Selbstsichernde Nahtmaterialien

Wird die Oberfläche der Nahtmaterialien mit **Widerhaken**

Widerhaken

versehen, die so angeordnet sind, dass sie beim Einstechen an der Oberfläche anliegen, bei entgegengesetzter Bewegung heraustreten, kann auf Knotung verzichtet werden [13, 14, 15]. Andere Autoren weisen auf die Zeitökonomie beim Einsatz dieser Materialien hin [16, 17, 18] und auf ein geringeres Gesamtkomplikationsrisiko bei (arthroskopischen) Knieoperationen [18].

### Nahtmaterialfarben

Gefärbtes Material kann dazu beitragen, während der Operation besser sichtbar zu sein, was bei Sehnennähten von Vorteil sein kann. Bei Intrakutannähten ist farbiges Nahtmaterial unvorteilhaft, da es durchscheinen kann und wie eine Hauttätowierung imponieren kann. Bei Hautnähten, die ja meist gezogen werden, erleichtert das Anfärben des Nahtmaterials durch Kontrastierung gegenüber der Haut das Fadenziehen.

#### Merke

Bei Intrakutannähten wird farbloses Nahtmaterial verwendet.

#### Farbmarkierung.

Die Farbkodierungen des Nahtmaterials auf den Verpackungen der verschiedenen Hersteller sind nicht kompatibel.

### Fadenstärke, -durchmesser, Kaliber

In Europa wird verbindlich nach der **Europäischen Pharmakopöe**

Europäischen Pharmakopöe

(Ph.Eur.) metrisch die Nahtmaterialstärke festgelegt. Auf den Nahtmaterialverpackungen finden sich zusätzlich Bezeichnungen der United States Pharmakopöe (USP) [19], die kein direktes Verhältnis zum Fadendurchmesser erkennen lassen (Tab. 1). Für USP gilt, je größer die Zahl des Nahtmaterials ist, desto kleiner ist der Durchmesser, z. B. ist ein 7‑0-Nahtmaterial kleiner als ein 4‑0-Nahtmaterial. Im Markt haben sich die historisch gewachsenen Stärkeangaben der USP weltweit durchgesetzt.

**Table 1:** 

Ph.Eur	USP	Durchmesserspanne in mm
0,01	12‑0	0,001–0,009
0,1	11‑0	0,010–0,019
0,2	10‑0	0,020–0,029
0,3	9‑0	0,030–0,039
0,4	8‑0	0,040–0,049
0,5	7‑0	0,050–0,069
0,7	6‑0	0,070–0,099
1	5‑0	0,100–0,149
1,5	4‑0	0,150–0,199
2	3‑0	0,200–0,249
2,5	2‑0	0,250–0,299
3	2‑0	0,300–0,349
3,5	0	0,350–0,399
4	1	0,400–0,499
5	2	0,500–0,599
6	3	0,600–0,699
7	5	0,700–0,799

*Ph.Eur.* Europäische Pharmakopöe, *USP* United States Pharmakopöe

Unabhängig davon, ob es sich um einen ein- oder mehrschichtigen Wundverschluss handelt, sollte die kleinste Fadenstärke bzw. der kleinste Durchmesser des Nahtmaterials gewählt werden, die bzw. der den jeweiligen Zweck erfüllt, um sowohl das **Gewebetrauma**

Gewebetrauma

bei jedem Nadeldurchgang als auch die Menge des zurückbleibenden Fremdmaterials zu minimieren. Materialien aus natürlichen und synthetischen Stoffen, aber auch aus Metall kommen zum Einsatz [1].

#### Merke

Benutze die kleinste Fadenstärke, die die Voraussetzungen erfüllt.

### Resorption

Die Begriffe Resorption und Absorption werden synonym verwendet. Die wichtigsten Merkmale für den biologischen Abbau und die Absorption von resorbierbarem Nahtmaterial sind das Festigkeits- und Massenverlustprofil sowie die Biokompatibilität der Abbaumaterialien. Bei **Nahtmaterial auf Proteinbasis**

Nahtmaterial auf Proteinbasis,

das durch proteolytische Enzyme und Phagozyten abgebaut wird, haften die Bakterien besser am Nahtmaterial [4]. Eine verstärkte Kapillarität erhöht ebenfalls das Infektionsrisiko, da sich die Mikroorganismen leichter bewegen und ausbreiten können [4].

Als **Resorptionszeit**

Resorptionszeit

wird die Zeitspanne benannt, in der das Material 50 % der Reißkraft des Knotens verliert. Von **Massenresorption**

Massenresorption

spricht man, wenn das gesamte Nahtmaterial vom Gewebe abgebaut ist.

#### Abbau durch Hydrolyse.

Synthetisches resorbierbares Nahtmaterial kann durch einen hydrolytischen Mechanismus über die Spaltung von Esterbindungen im Polymergerüst abgebaut werden. Sie werden durch **natürliche Stoffwechselprozesse**

natürliche Stoffwechselprozesse

fast rückstandsfrei abgebaut [20]. Wenn die Moleküle hydrophob sind, wird ihre Hydrolyse verzögert und ihre Absorptionszeit verlängert [20].

Eindringendes Wasser in das Nahtmaterial zerstört die Polymerstruktur des Fadens. Die Aufrechterhaltung des Gleichgewichts zwischen schneller Absorption und der Verlängerung der Zugfestigkeit wurde durch Behandlungen und chemische Strukturierung unterstützt, die die Absorptionszeit verlängern [1]. Die Art des Abbaus, den das Material erfährt, seine Kapillarität und seine physikalische Konfiguration beeinflussen das Infektionsrisiko [4]. Bei der Hydrolyse kommt es zu einer geringeren Gewebereaktion als beim enzymatischen Abbauprozess [1].

#### Enzymatischer Abbau.

Beim Wundverschluss mit resorbierbarem Nahtmaterial nimmt die Zugfestigkeit in den ersten Wochen allmählich und meist linear ab. Es findet physiologisch eine Gewebe- und Zellreaktion statt, um Zelltrümmer und abbaubare Anteile des Nahtmaterials zu entfernen. Diese Phasen können durch Infektionen und Eiweißmangel beeinträchtigt werden, wobei die Zugfestigkeit schneller verloren geht und eine Wunddehiszenz klinisch sichtbar werden kann. Nach dem primären Zusammenhalt des Gewebes werden diese Nahtmaterialien durch enzymatische oder hydrolytische Prozesse im Gewebe abgebaut. Die **Abbaurate**

Abbaurate

variiert in Abhängigkeit vom Material, der Lokalisation und patientenabhängigen Faktoren (Tab. 2). Durch diese Prozesse werden Zugfestigkeit und Reißkraft konstant meist nach einer vom Material abhängigen Latenzzeit vermindert.

**Table 2:** 

Fadenstärke	Haut	Tiefere Schicht	Spezieller Einsatz
1			Faszie, Sehne, Ligamente Bein
0			Bauchwand
2‑0	Fußsohle		Gefäßligatur
3‑0	Fuß	Rücken, Thorax, Abdomen	Beugesehnen, Muskel
4‑0	Extremitäten, Fuß(-Rücken), Kopfhaut, Thorax	Extremitäten, Fuß, Kopfhaut	
5‑0	Hand, Stirn, Kopfhaut	Hand, Gesicht, Stirn, Nase	
6‑0	Gesicht, Augenlid, Nase, Lippe, Ohr		
7‑0	Augenlid, Lippe, Gesicht, Lid		
8‑0			Nervennaht
9‑0			Mikrochirurgie Nerven, Gefäße
10‑0			Mikrochirurgie

## Nahtmaterialien

Bei den Nahtmaterialien wird unterschieden zwischen natürlichen und synthetischen resorbierbaren Materialien. Die European Association of the Surgical Suture Industry (**EASSI**

EASSI)

hat für die verschiedenen Materialien und Spezifikationen Vorgaben erlassen, die in DIN EN ISO 15223‑1 dokumentiert sind ([21], Tab. A1).

### Resorbierbare Materialien

#### Nahtmaterial aus natürlichen organischen Ausgangsmaterialien

Natürliche Nahtmaterialien können in organische resorbierbare Materialien (aus Kollagen gewonnen, **Katgut**

Katgut)

und nicht resorbierbare (Seide, Zwirn) unterteilt werden. Sie werden seltener verwendet, da sie tendenziell eine **stärkere Gewebereaktion**

stärkere Gewebereaktion

hervorrufen. **Nähseide**

Nähseide

wird jedoch immer noch regelmäßig bei der Sicherung von chirurgischen Drainagen verwendet.

##### Kollagen.

In der Vergangenheit wurden organische Nahtstoffe gewählt, zu dieser ältesten, ursprünglichen Gruppe gehörten Katgut, mit Chromsalz gegerbtes Chromkatgut und **Kollagenbänder**

Kollagenbänder

[22].

Diese wurden zuerst aus der Submukosa des Schafdarms und später der Serosa des Rinderdarms gewonnen [22]. Dieses Material wurde gezwirnt, war also multifilamentär, oder wurde beschichtet, z. B. mit Silikon [22]. Katgut aus Rindern wurde Anfang der 2000er-Jahre vom Markt genommen. Rinderkollagen von BSE(bovine spongiforme Enzephalopathie)-freien Rindern wird inzwischen wieder angeboten.

##### Seide.

Die Grundsubstanz der chirurgischen Seidenfäden wird aus dem Kokon der Seidenraupenlarve gewonnen und besteht aus Fibroin der Rohseidenfaser, dem die Kittsubstanz Sericin entzogen wurde [22]. Seidenfäden sind geflochten und können durch Beschichtungen, z. B. Bienenwachs oder Silikon, zum **pseudomonofilen Nahtmaterial**

pseudomonofilen Nahtmaterial

werden [22].

##### Zwirne.

Zwirnfäden – in Europa aus Flachs, in den USA aus der Zellulose von Baumwolle hergestellt – werden immer in multipler Konfiguration angeboten [22]. Diese Materialien werden in Orthopädie und Unfallchirurgie bei uns kaum eingesetzt.

### Synthetische resorbierbare Materialien

Sie bestehen aus künstlich hergestellten Materialien. Die Eigenschaften sind meist konstanter als bei den natürlichen Nahtmaterialien, insbesondere was den Verlust der Zugfestigkeit und die Absorption betrifft.

Sie bestehen meist aus **chemischen Polymeren**

chemischen Polymeren,

die durch Hydrolyse resorbiert werden. Diese Materialien haben durchweg eine geringere Gewebereaktion als natürliche Stoffe. Bei den resorbierbaren synthetisch hergestellten Nahtmaterialien finden sich folgende Monomaterialien oder auch Copolymere (s. auch Tab. A1 und A2 online).

#### Lactomer-Copolymer/Polyglactin 910.

Dieses Nahtmaterial ist ein **geflochtenes Multifilamentnahtmaterial**

geflochtenes Multifilamentnahtmaterial,

das mit einem Copolymer aus Lactid (10 %) und Glycolid (90 %) besteht und teilweise beschichtet angeboten wird mit Polyglactin 370 plus Calciumstearat. Die wasserabweisende Eigenschaft von **Lactid**

Lactid

verlangsamt den Verlust der Zugfestigkeit, und die Bauschigkeit von Lactid führt zu einer schnellen Resorption des Nahtmaterials, sobald die Zugfestigkeit verloren geht. Hiermit können unterschiedliche Eigenschaften in einem Nahtmaterial verbunden werden. **Glycolid**

Glycolid

sorgt für eine hohe anfängliche Zugfestigkeit, wird aber im Gewebe schnell hydrolysiert [23]. Lactid hat eine langsamere und kontrolliertere Hydrolyserate und somit auch einen langsameren Zugfestigkeitsverlust [23]. Eine Beschichtung aus einer resorbierbaren Mischung auf dem Nahtmaterial aus Caprolacton-Glycolid-Copolymer und Calciumstearyl-Lactylat [24, 25] erzeugt eine Reduzierung der Oberflächenreibung, eine präzise Knotenplatzierung und ein reibungsloses Ligieren. Die Zugfestigkeit des Polyglactin 910-Nahtmaterials liegt am Tag 14 nach der Implantation bei ca. 65 %. Durch Hydrolyse ist nach 56 bis 120 Tagen vollständig erfolgt (s. Tab. A2 online). Diese Fäden verursachen nur eine minimale Gewebereaktion. Sie werden bei der allgemeinen Weichteilapproximation und Gefäßligatur eingesetzt.

#### Polyglycolsäure.

Polyglycolsäure (PGS) wird mit Polycaprolat oder Polyol beschichtet. Diese wird ungefärbt und farbig in violett angeboten. Das Material wird meist geflochten angeboten. Zugfestigkeit und Resorptionsschnelligkeit verhalten sich ähnlich wie bei Polyglactin 910 nach 90 bis 120 Tagen.

#### Poliglecaprone 25.

Poliglecaprone 25 ist ein **monofiles Nahtmaterial**

monofiles Nahtmaterial.

Es ist ein Copolymer aus Glycolid und ε‑Caprolacton. Die Biegsamkeit ist sehr hoch, was eine einfache Handhabung ermöglicht. Die Zugfestigkeit ist anfänglich hoch, 50–60 % am Tag 7 nach der Implantation und nimmt am Tag 21 ab. Die Resorption ist nach 91 bis 119 Tagen abgeschlossen. Poliglecaprone 25-Nähte werden für subkutane Nähte und Weichteilapproximationen und Ligaturen verwendet. Poliglecapron 25-Nähte verursachen signifikant weniger Fadenextrusion als Polyglactin 910 [26]. Die Knotenfestigkeit wird als gut eingestuft. Es wird für schnell heilende Gewebe genutzt. Die Gewebereaktion ist gering.

#### Polydioxanon.

Dieses monofile Nahtmaterial ist aus Polyester hergestellt, bietet eine **längere Wundunterstützung**

längere Wundunterstützung

und ruft nur eine geringe Gewebereaktion hervor. Die Zugfestigkeit beträgt 70 % an Tag 14 und 25 % an Tag 42. Die Wundunterstützung bleibt bis zu 6 Wochen erhalten.

Die Resorption durch Hydrolyse ist in den ersten 90 Tagen minimal und innerhalb von 6 Monaten im Wesentlichen abgeschlossen. Wie andere monofile Nahtmaterialien hat Polydioxanon eine geringe Affinität zu Mikroorganismen. Es wird für die Approximation von Weichteilen verwendet.

#### Poly(glycolid)trimethylencarbonat.

Polytrimethylencarbonat hat ähnliche Zugfestigkeit und Absorption wie Polydioxanon.

#### Glycomer 631.

Polymer aus Glysolid, Dioxanon, Trimethylen-Karbonat.

#### Polygytone 6211.

Polymer aus Glysolid, Caprolacton, Trimethylen-Karbonat und Lactid.

### Nichtresorbierbare Materialien

Diese Materialien verfügen über eine **gute Festigkeit**

gute Festigkeit,

können aber im Gewebe Fremdkörperreaktionen hervorrufen. **Gewebereaktionen**

Gewebereaktionen

führen dabei im Verlauf zu Ummantelung des Nahtmaterials. Diese Abkapselung erfolgt durch Fibroblasten. Auch Makrophagen und Riesenzellen finden sich in der Umgebung [1]. Nichtresorbierbare Materialien können durch Beschichtung eine Friktionsverbesserung und/oder auch eine geringere Kapillarität erhalten.

Diese nichtresorbierbaren Fäden werden einerseits in langsamer heilenden Geweben eingesetzt, aber auch dort, wo starker Halt gefordert wird, z. B. Sehnen.

Unter **Verfallszeit**

Verfallszeit

versteht man bei nichtresorbierbarem Nahtmaterial die Zeit, in der durch Degradation das Material in Abschnitte zerfällt.

#### Natürliche nichtresorbierbare Materialien

Zu den natürlichen, nichtresorbierbaren Nahtmaterialien gehören chirurgische Seide, Zwirn und Baumwolle.

Sie werden seltener verwendet, da sie tendenziell eine stärkere Gewebereaktion hervorrufen. Nähseide wird in einigen Regionen immer noch regelmäßig bei der Sicherung von chirurgischen Drainagen verwendet.

##### Chirurgische Seide.

Aus Rohseide, also den Kokons der Seidenraupen, wird dieses Nahtmaterial gesponnen. Eine Beschichtung kann mit Wachs, z. B. Bienenwachs oder Silikon, erfolgen. Seide lässt sich gut knoten, und vielen gilt die Handhabung mit Seide als der Standard.

Bei längerer Proteolyse kann Seide vom Gewebe innerhalb von 2 Jahren resorbiert werden. Die Zugfestigkeit nimmt mit der Feuchtigkeitsaufnahme ab und geht nach 1 Jahr verloren. Das Hauptproblem bei Seidenfäden ist eine **akute Entzündungsreaktion**

akute Entzündungsreaktion.

Das umliegende Gewebe verkapselt das Nahtmaterial durch faseriges Bindegewebe. Seidenfäden haben sehr gute Knüpfeigenschaften, sind geschmeidig und haben damit eine gute Handhabung. Seide hat eine hohe Kapillarität und ermöglicht einen hohen interfilamentären Bakterientransport [11].

##### Chirurgische Baumwolle.

Chirurgische Baumwolle wird in den deutschsprachigen Ländern so gut wie nicht eingesetzt. Sie wird hier nur der Vollständigkeit halber genannt. Dieses Nahtmaterial wird aus gedrehten, langstapeligen Baumwollfasern hergestellt. Die Zugfestigkeit beträgt 50 % innerhalb von 6 Monaten und 30–40 % nach 2 Jahren. Die nichtresorbierbare Baumwolle wird im Körpergewebe eingekapselt. Als nachteilig angesehen werden Kapillarität, Gewebereaktion und Bakteriophilie.

#### Natürliche anorganische Ausgangsmaterialien

Meist werden **Edelstahle**

Edelstahle

verwendet. Tantal ist im Gebrauch. Korrosionsbeständige Metalle sind notwendig, da durch die Körperflüssigkeiten die Metalloberflächen angegriffen werden, was sogar bei hochwertigen Titanimplantaten als **Metallose**

Metallose

imponieren kann. Nahtmaterial aus Stahl wird monofil oder polyfil angeboten.

#### Synthetische nichtresorbierbare Materialien

Polyester und Polyolefine sind 2 Vertreter dieser Gruppe.

##### Polyester.

Das **unauflösbare Polyestermaterial**

unauflösbare Polyestermaterial

besteht aus den Rohstoffen Polyethylenterephatalat oder Polybutylenterephtalat. Es werden monofile, multifile, geflochtene und pseudomonofile Fäden angeboten. Sie werden mit Polytetrafluoräthylen, Polytetramethylenadipat, Silikonkautschuk, Äthylcellulose, Wachs oder Polyolefine beschichtet, um bei den geflochtenen Konfigurationen eine pseudomonofile Eigenschaft zu erreichen [22]. Monofile Fäden weisen keinen interfilamentären Bakterientransport auf [11]. Polyester hat eine hohe Knotenzugfestigkeit, gute Flexibilität und geringe chemische Degradation.

##### Polyolefine.

Sie bestehen aus Polypropylen oder Polyäthylen und werden durchweg als monofiles Material angeboten [22]. Die Oberfläche ist so glatt, dass eine Beschichtung nicht erforderlich ist. Polypropylen hat einen ausgezeichneten Gewebewiderstand und **Stabilität**

Stabilität.

##### Polyamide.

Nahtmaterialien aus der Gruppe der Polyamide werden nicht resorbiert oder absorbiert, sie zerfallen nach längerer Liegezeit. Sie werden hergestellt aus Polyamid 6.6 (Hexamethylendiamin und Adipinsäure) [Nylon], oder aus Polyamid 6 Aminocapronsäure. Sie werden in folgenden Konfigurationen angeboten: monofil, geflochten, multifil, pseudomonofil. Als Beschichtung dient Polyamid 6 oder eine Imprägnierung mit Bienenwachs [22]. Die Hauptnachteile von Polyamid-Nahtmaterial sind seine **schlechten Handhabungseigenschaften**

schlechten Handhabungseigenschaften

und seine Knotensicherheit [27].

### Auswahl von Material und Fadenstärke

Beim Verschluss von Wunden oder chirurgischen Zugängen werden üblicherweise in der Subkutis und Subdermis resorbierbare Nähte und für den Hautverschluss folgende monofile nichtresorbierbare Nähte verwendet:

- oberflächliche Läsionen im Gesicht: 6‑0,
- andere oberflächliche Hautläsionen:
  - in Bereichen mit niedriger Hautspannung: 5‑0,
  - in Bereichen mit höherer Hautspannung: 4‑0.

Differenziert man nach Fadenstärke, schlagen die Autoren in [28] folgende Auswahl vor (s. Tab. 2).

Die vorgeschlagenen **Fadenstärken**

Fadenstärken

müssen dem Alter, der Körperkonstitution und dem Gewebezustand angepasst werden. Beim Wundverschluss wird das Material abhängig von der Schicht verwendet: in der Tiefe eher resorbierbar und polyfil, in der Haut selbst monofil nicht resorbierbar (Tab. 3 und 4).

**Table 3:** 

Schicht		Resorption		Aufbau		Material	
Tief subdermal	Oberflächlich	+		Monofil	Polyfil	I	II
x		x			x^a^	Polymilchsäure	Polyglactin 910^b^
x		x		x		Polyglecapron 25	Polydioxanon
	x		x	x		Nylon	(Poly )Propylen

^a^Nicht bei Infektionsrisiko

^b^Häufig eingesetzt

**Table 4:** 

Gewebe	Nahttechnik		Resorption		Aufbau		Material
Gefäß	Fortlaufend	Einzelknopf	+		Monofil	Polyfil	I
Gefäß	x			x	x		Polyvinylidenfluorid
Gefäß	x			x	x		Polypropylen
Gefäß	x	x		x	x		Poly-p-dioxanon
Umstechung			x			x^a^	Polyglycolsäure
Umstechung				x		x^a^	Polyester
Muskulatur			x			x^a^	Polyglactin 910
Weichgewebe			x			x^a^	Polyglactin 910
Weichgewebe			x		x		Poligecaprone 25
Weichgewebe			x		x		Polydioxane
Faszie/Kapsel			x		x		Posterior cruchiate ligament (PCL)
Faszie/Kapsel			x		x		Polydioxane
Subkutan			x			x^a^	Polyglactin 910
Haut intrakutan	x			x	x		Polyamid 6‑6.6
Haut intrakutan	x		x		x		Glycolid-ε-Caprolacton-Mischpolymerisat
Haut intrakutan	x		x		x		Polyglycolsäure
Haut		x		x	x		Polypropylen
Haut		x		x	x		Polyamid 6‑6.6
Haut		x	x		x		Poligecaprone 25
Haut		Donati, Allgöwer		x	x		Polyamid 6‑6.6
Haut		Donati, Allgöwer	x		x		Polyglycolsäure
Haut		Donati, Allgöwer		x	x		Polypropylen
Haut		Donati, Allgöwer	x			x^a^	Glycolid-ε-Caprolacton-Mischpolymerisat
Bänder, Sehnen			x		x		Polydioxane

^a^Nicht bei Infektionsrisiko

### Beschichtung von Nahtmaterial mit Trägerstoffen

#### Antibiotikabeschichtung.

Metaanalysen konnten zeigen, dass die Anwendung von mit **Triclosan**

Triclosan

antimikrobiell beschichtetem Nahtmaterial die Inzidenz von „surgical site infections“ nach sauberen, sauber-kontaminierten und kontaminierten Operationen reduzierte [29, 30]. In Orthopädie und Unfallchirurgie werden diese Fäden selten genutzt, da keine Evidenz vorliegt [31]. Neuere Beschichtungen auf Chlorhexidin-Basis [32] oder Octenidindihydrochlorid [33] sind in der Entwicklung.

#### m(„messenger“)RNA(Ribonukleinsäure)-Beschichtung.

Experimentelle Untersuchungen lassen für die Zukunft die Entwicklung von mRNA-beschichtetem Nahtmaterial beim chirurgischen Wundverschluss in den Fokus rücken. Zellen im Wundbereich könnten direkt übertragen werden, wodurch die Wundheilung beschleunigt und verbessert werden könnte [10].

### Handhabung von Verpackung und Nahtmaterial

Obwohl es für Verpackungen und Beschriftung ISO-Normen gibt (ISO 20417) [34] ISO Nr. 15223 [21], finden sich auf dem Markt **keine standardisierten Verpackungen**

keine standardisierten Verpackungen.

Dies bedeutet für den Anwender, dass er je nach Hersteller sich über die Handhabung der Verpackungen informieren muss.

Die primäre Innenverpackung liegt in einer äußeren, meist durchsichtigen **Peel-Back-Verpackung**

Peel-Back-Verpackung.

Die Sterilität bleibt erhalten, bis sie geöffnet wird oder das Verfallsdatum erreicht ist.

**Figure 3:**
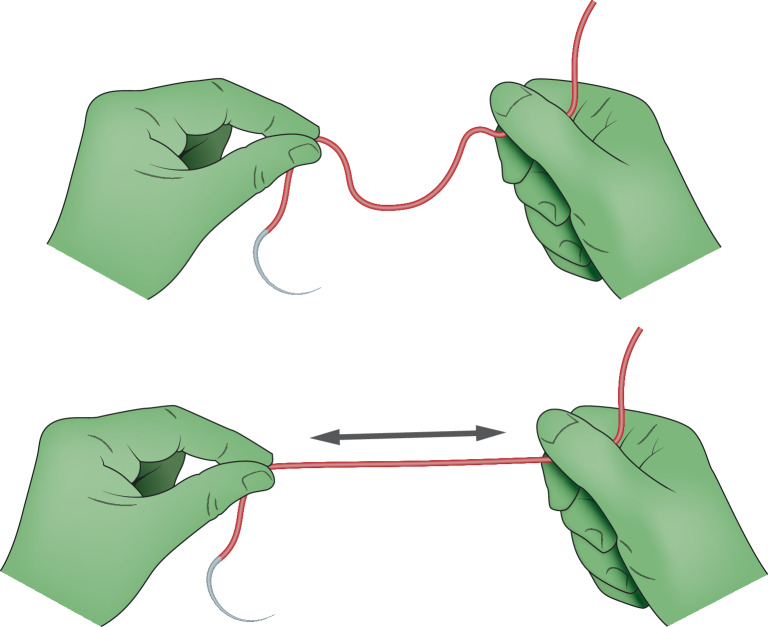

Die **sterilen Inhalte**

sterilen Inhalte

der Verpackungen werden von der OP-Pflegekraft oder dem Arzt durch Aufreißen der Umverpackung angereicht. Die sterile Verpackung lässt dann sich über Laschen öffnen. Der Inhalt wird nach Umklappen einer Lasche geöffnet. Die Nadel liegt frei und kann mit dem Nadelhalter in der Verpackung gefasst werden (s. hierzu [9]). Nach Aufklappen des Fadenträgers (Wickelträgers) wird die freigelegte Nadel mit anhängendem Faden entnommen. Diese kann vorsichtig gestreckt werden, um den Fadendrill zu vermindern. Hierbei sollte der Faden nicht zwischen Nadel und Fadenende, sondern nur innerhalb des Fadens gestreckt werden (Abb. 3). Bei Ligaturen oder nadelfreien Fäden liegt das Material dementsprechend in der Verpackung.

## Knotenanzahl

Bei geflochtenem Nahtmaterial ist es aufgrund der größeren Reibehaftung normalerweise ausreichend, **3 Knoten**

3 Knoten

zu legen und anzuziehen, wobei einer gegenläufig sein sollte.

Bei monofilem Nahtmaterial ist die Knotenzahl zu erhöhen, da die Reibehaftung aufgrund der glatten Oberfläche geringer ist. Hier gilt im Allgemeinen die Regel Fadenstärke in USP plus mindestens 1 zusätzlicher Knoten.

## Chirurgische Knotentechniken

Eine sichere chirurgische Knotentechnik ist eine Grundlage für den Erfolg einer chirurgischen Maßnahme. Der fertige Knoten sollte fest, straff und nicht rutschend sein.

Bei der **Hautnaht**

Hautnaht

liegt der Knoten nicht in der Inzisionslinie, um das Infektionsrisiko zu verringern.

### Merke

Bei der Hautnaht liegt der Knoten immer seitlich der Inzisionslinie.

**Figure 4:**
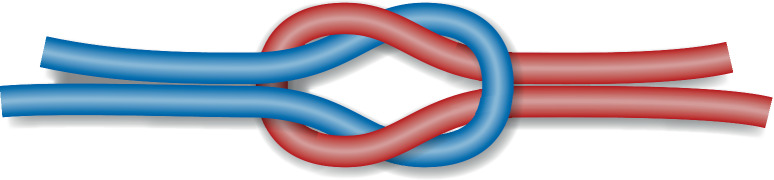

Der chirurgische Knoten ist ein **modifizierter Riffknoten**

modifizierter Riffknoten

mit einer zusätzlichen Drehung im ersten Wurf, die die Zugfestigkeit des Knotens erhöht. Es ist darauf zu achten, dass der Knoten flach aufliegt; dies kommt beim sog. Samariter-Knoten oder auch Kreuzknoten genannt zustande (Abb. 4).

Die Knoten sollten klein sein. Am Schluss werden die Enden kurz geschnitten (2–4 mm).

### Überkreuzter Knoten in Einhandtechnik

**Figure 5:**
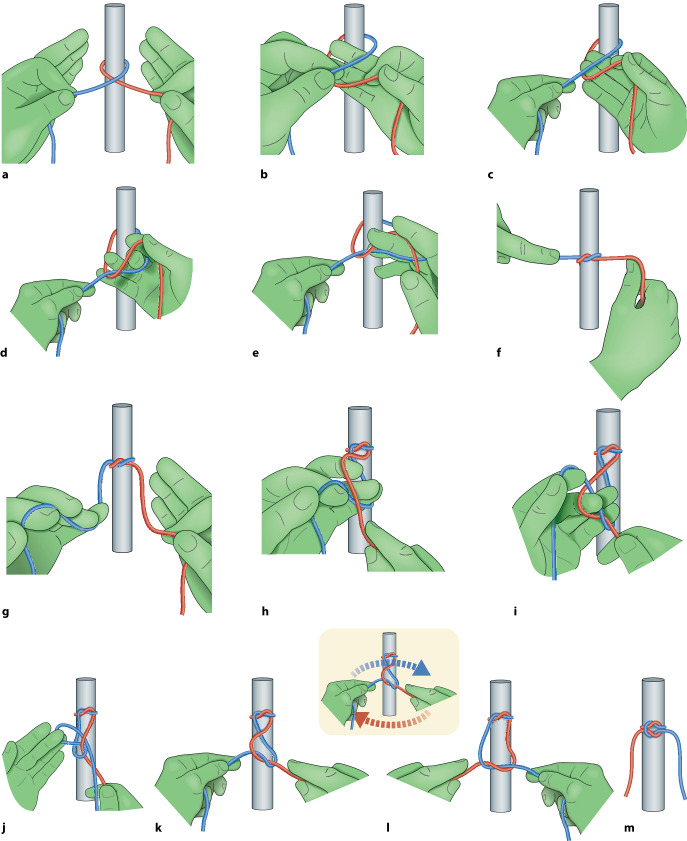

**Figure 6:**
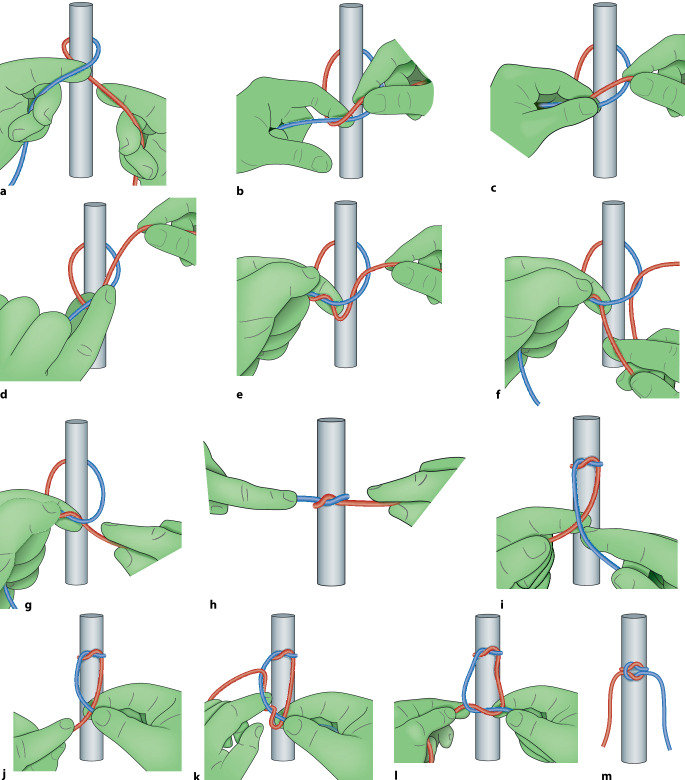

Um Gegenläufigkeit zu erreichen, wird normalerweise mit beiden Händen geknotet (Abb. 6a–m), einhändige Technik kann in manchen Situationen aber auch erforderlich werden (Abb. 5a–d). Die Legenden geben die Details wieder.

### Überkreuzter Knoten in Zweihandtechnik

Dieser Knoten ist einfach zu knoten und hat einen guten Knotensitz (s. auch Abb. 6e–m) Die Legenden geben die Details wieder.

## Fadenentfernung

### Zeitpunkt des Fadenzugs

Hautfäden werden am zügigsten im Kopf-Hals-Bereich und bei Kindern gezogen. Bei Immunsuppression, z. B. Cortison-Einnahme, Diabetes mellitus sollte der Fadenzug prolongiert wegen der verzögerten Wundheilung erfolgen (s. auch Tab. 5).

**Table 5:** 

Lokalisation	Erwachsene [Tage]	Kinder [Tage]	Bemerkung
Kopfhaut	10	7	
Gesicht	5	4	Danach Klebestrip
Augenbraue	5	4	
Augenlid	3	3	
Ohr	5	4	
Lippe	5	4	
Hals	5	4	
Obere Extremitäten	10–12	8–10	
Hand	12	8–10	
Untere Extremität	12–14	8–10	
Fuß	12–14	8–10	
Rücken	12–14	10–12	

### Fadenzug

Zum Fadenzug eignen sich **verschiedene Techniken**

verschiedene Techniken.

**Figure 7:**
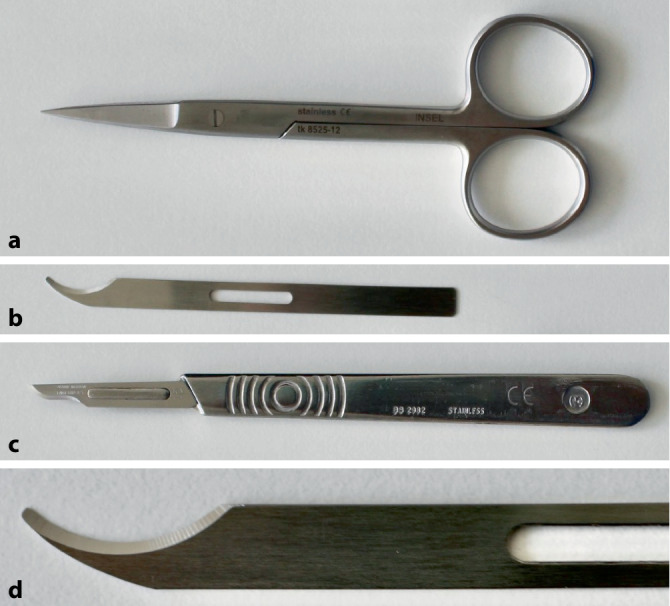

**Figure 8:**
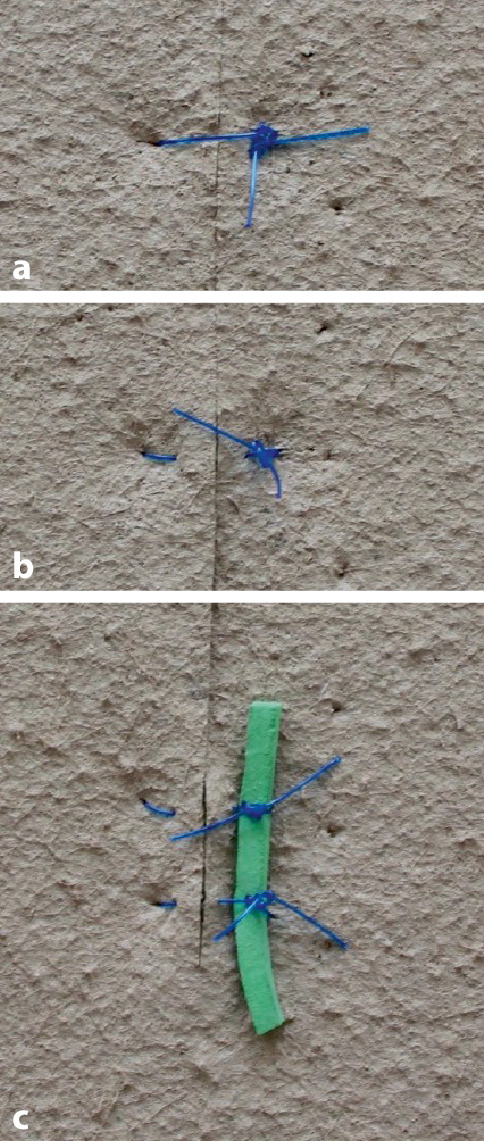

**Figure 9:**
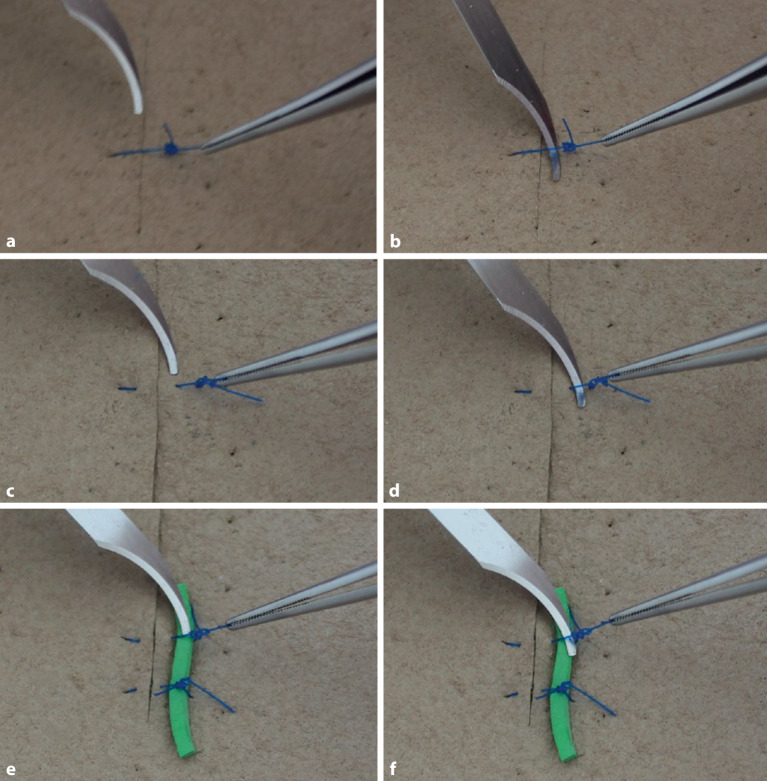

Ein Ende der Naht wird mit einer anatomischen Pinzette gefasst und vorsichtig angehoben oder zu einer Seite der Wunde gezogen. Bei Verwendung normaler Skalpellklingen wird beachtet, dass die Schneide hautabgewandt ist. Identisches Vorgehen kann auch mit einer spitzen Schere oder einem speziellen Fadenmesser erfolgen (Abb. 7, 8, 9, 10 und 11).

**Figure 10:**
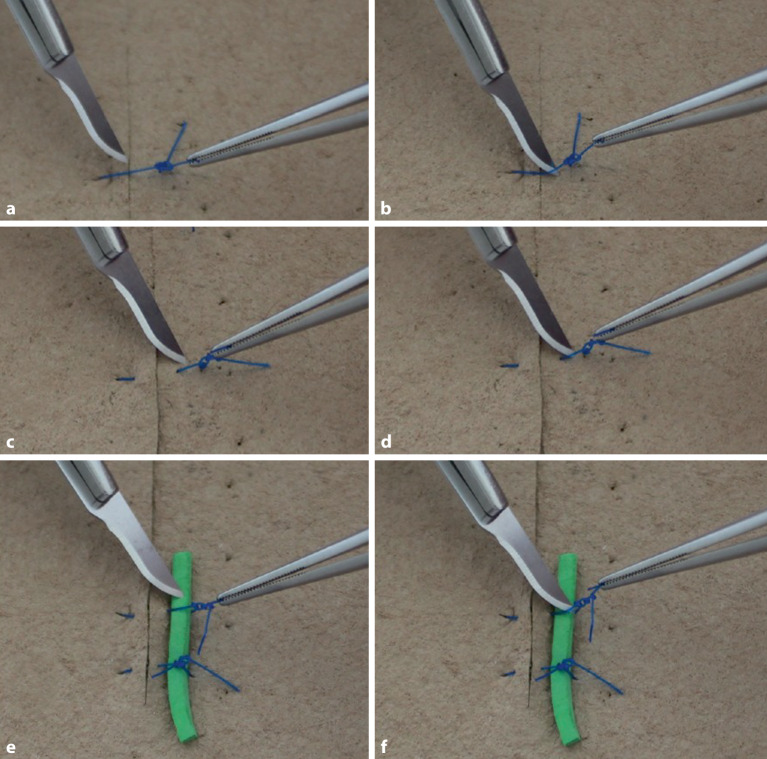

**Figure 11:**
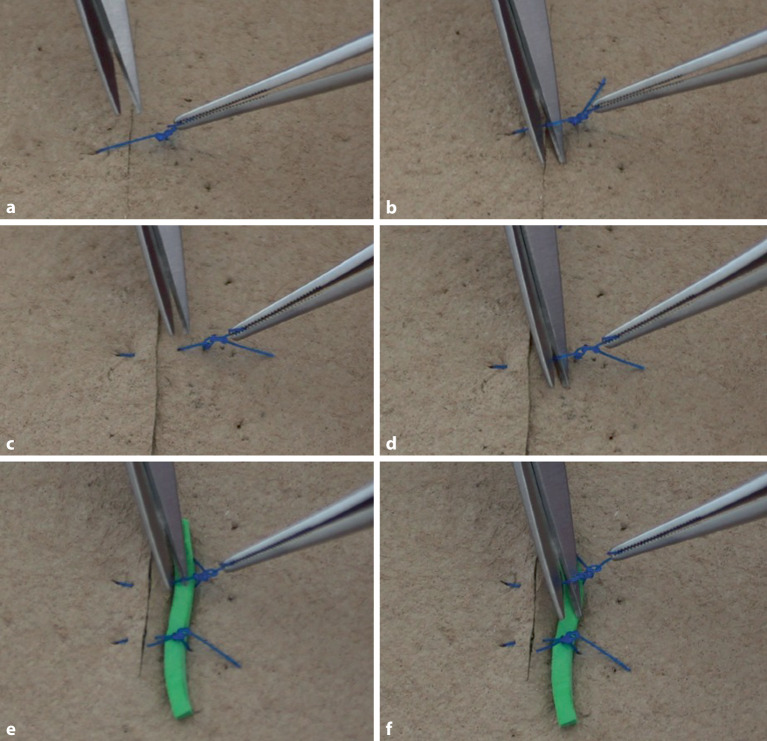

### Supplementary Information






## CME-Fragebogen

Welche Aussage bezüglich des Knotens bei einer Hautnaht ist richtig?

Der Knoten sollte direkt über der Inzision liegen.

Der Knoten sollte seitlich der Inzision liegen.

Der Knoten sollte abwechselnd links und rechts seitlich der Inzision liegen.

Der Knoten sollte möglichst weit entfernt von der Inzision liegen.

Der Knoten über der Inzisionslinie verringert das Infektionsrisiko.

Welche Aussage bezüglich des Knotens mit Nahtmaterial ist richtig?

Die Knoten halten durch Reibehaftung.

Knoten halten schlechter bei geflochtenem Nahtmaterial.

Die Knoten sollten nicht flach auf der Haut liegen.

Die Enden der Knoten sollten mindestens 1,5 cm lang sein.

Bei monofilem Nahtmaterial werden grundsätzlich weniger Knoten verwendet.

Durch welchen Faktor wird die Qualität der Naht *nicht* beeinflusst?

Entstehungsart der Wunde (elektiv oder traumatisch)

Lokalisation der Wunde

Hautdicke/Hautbeschaffenheit

Spannung der Weichteile

Hautdesinfektionsmittel

Welche Eigenschaft des Nahtmaterials spielt bei der Auswahl *keine* Rolle?

Reißfestigkeit

Kapillarität

Biegsamkeit

Resorptionsfähigkeit

Schneidbarkeit des Fadens

Was trifft bei geflochtenem Nahtmaterial zu?

Dieses hat aufgrund der Oberflächenrauigkeit eine niedrige Reibehaftung.

Mit einem steilen Eintrittswinkel zur Oberfläche wird die Sägewirkung des Fadens im Gewebe verringert.

Eine Dochtwirkung auf dem Boden von Kapillarität kann nicht auftreten.

Die Dochtwirkung ist bei gezwirntem geringer als bei geflochtenem Nahtmaterial.

Eine Beschichtung erhöht allgemein die Reibekraft dieses Materials.

Welche Aussage bezüglich Nahtmaterialfarben und -stärke ist richtig?

Bei der Fadenstärke nach United States Pharmakopöe (USP) kennzeichnet eine höhere Zahl einen dickeren Faden.

Gefärbte Nahtmaterialien bieten sich insbesondere für Subkutanfäden an.

Farbmarkierungen auf den Verpackungen verschiedener Hersteller sind vergleichbar.

Die gewählte Fadenstärke ist von der Anzahl der Wundschichten abhängig.

Je dünner ein Faden desto kleiner die Zahl nach Europäischer Pharmakopöe (Ph.Eur.).

Welche Aussage bezüglich resorbierbaren Nahtmaterials trifft zu?

Durch enzymatischen Abbau nimmt die Zugfestigkeit in den ersten Wochen allmählich ab.

Synthetische Nahtmaterialien werden durch Enzyme resorbiert.

Seide ist ein typisches organisches resorbierbares Nahtmaterial, das innerhalb von 3 Monaten resorbiert wird.

Synthetische resorbierbare Nahtmaterialien werden grundsätzlich nicht beschichtet verwendet.

Synthetische resorbierbare Nahtmaterialien werden allgemein durch Kollagenasen abgebaut.

Welche Fadenstärke sollte entsprechend dem anatomischen Gebiet nach United States Pharmakopöe (USP) gewählt werden?

Beim Hautverschluss mit niedriger Hautspannung können 10-0-Fäden verwendet werden.

Faszien und Sehnen werden mit Fadenstärke 7‑0 genäht.

Wunden in Gesicht, an Augenlid, Nase, Ohr werden mit 6‑0 oder 7‑0 genäht.

Am Fuß wird in der Regel die Fadenstärke 1 verwendet.

In der Mikrochirurgie werden Fäden der Stärke 3‑0 oder 4‑0 eingesetzt.

Welche Aussage ist bezüglich Nahtmaterial und Anwendungsgebiet richtig?

Bei Gefäßnähten ist resorbierbares Nahtmaterial üblich.

Intrakutannähte können mit resorbierbarem oder nichtresorbierbarem Material erfolgen.

Bei Gefäßnähten ist der Einsatz von polyfilen Fäden üblich.

Bei der Naht von Muskeln ist nichtresorbierbares Nahtmaterial üblich.

Für das Nähen des Periostes gibt es kein geeignetes Nahtmaterial.

Was trifft für chirurgische Seidennähte zu?

Die Zugfestigkeit bleibt immer konstant, da Seide nicht resorbiert wird.

Bei längerer Protolyse kann Seide vom Gewebe innerhalb von 2 Jahren resorbiert werden.

Seide eignet sich v. a. bei infizierten Wunden, da sie einen hohen antiinterfilamentären Effekt hat.

Seide sollte heute nicht mehr verwendet werden.

Da chirurgische Seide aus den Kokons der Seidenraupen gewonnen wird, braucht sie keine spezielle Beschichtung zur Verbesserung der Gleitfähigkeit.
