# Supplementary material for: Surgical suture material—fundamentals
Source: Oper Orthop Traumatol. 2023 Aug 21;35(5):298–316. [Article in German] doi: 10.1007/s00064-023-00812-y (PMC10520208; doi:10.1007/s00064-023-00812-y)
Supplement: Supplementary file 1 [file 64_2023_812_MOESM1_ESM.pdf]

Tabelle 1

Fadenmaterial Symbol	engl. Bedeutung	deutsche Bedeutung
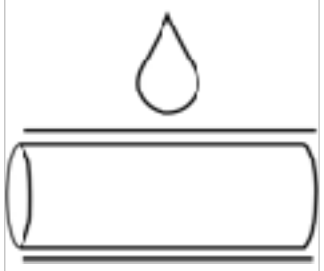	undyed / monofilament / nonabsorbable / coated	ungefärbt / monofil / nicht resorbierbar / beschichtet
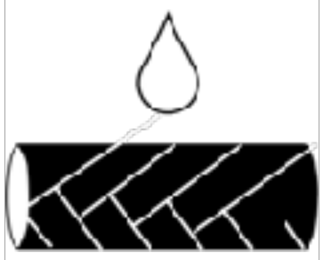	dyed / braided / nonabsorbable	eingefärbt / geflochten / nicht resorbierbar
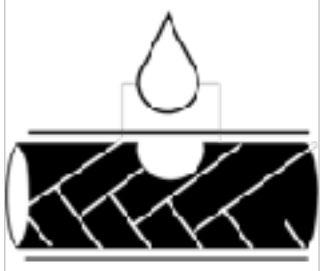	dyed / braided / coated / absorbable	gefärbt / geflochten / beschichtet / resorbierbar
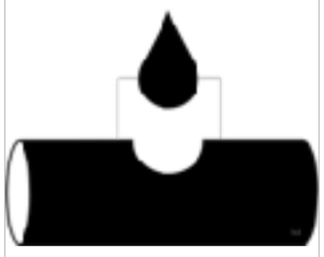	dyed / monofilament / absorbable	eingefärbt / monofil / resorbierbar
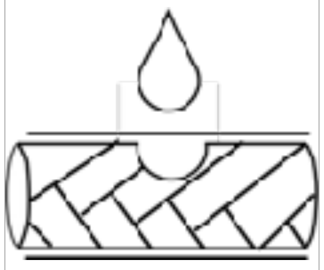	undyed / braided / coated / absorbable	ungefärbt / geflochten / beschichtet<br>beschichtet / resorbierbar
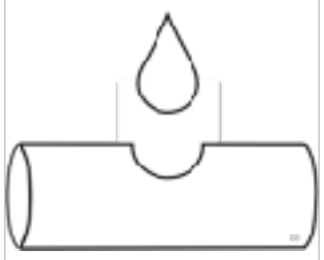	undyed / monofilament / absorbable	ungefärbt / monofil / resorbierbar

Fadenmaterial Symbol	engl. Bedeutung	deutsche Bedeutung
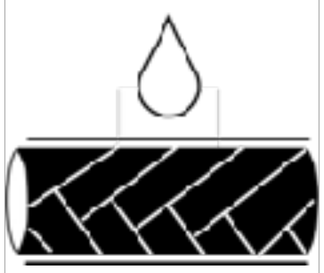	dyed / braided / coated / nonabsorbable	gefärbt / geflochten / beschichtet / nicht resorbierbar
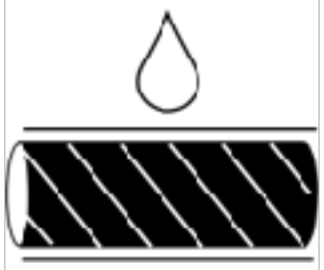	dyed / twisted / coated nonabsorbable	gefärbt / gezwirnt / beschichtet / nicht resorbierbar
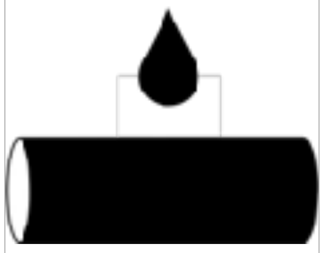	dyed / monofilament / nonabsorbable	gefärbt / monofil / nicht resorbierbar
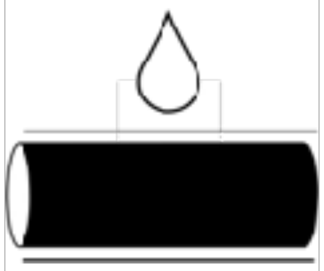	dyed / monofilament / coated nonabsorbable	eingefärbt / monofil / beschichtet / nicht resorbierbar
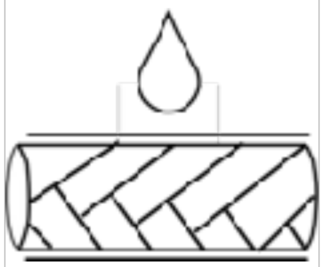	undyed / braided / coated / nonabsorbable	ungefärbt / geflochten / beschichtet / nicht resorbierbar
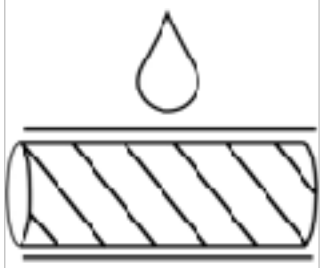	undyed / twisted / nonabsorbable	ungefärbt / gezwirnt / nicht resorbierbar
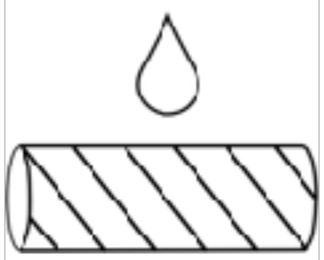	undyed / twisted / nonabsorbable	ungefärbt / gezwirnt / nicht resorbierbar

Fadenmaterial Symbol	engl. Bedeutung	deutsche Bedeutung
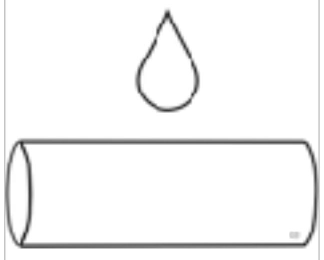	undyed / monofilament / nonabsorbable	ungefärbt / monofil / nicht resorbierbar
modifiziert nach EUROPÄISCHE VEREINIGUNG DER HERSTELLER VON CHIRURGISCHEM NAHTMATERIAL E. V., BERLIN, 10117 Berlin, Reinhardtstr. 29 b<br>EASSI – The European Association of the Surgical Suture Industry		
