# Supplementary material for: Surgical suture material—fundamentals
Source: Oper Orthop Traumatol. 2023 Aug 21;35(5):298–316. [Article in German] doi: 10.1007/s00064-023-00812-y (PMC10520208; doi:10.1007/s00064-023-00812-y)
Supplement: Supplementary file 2 [file 64_2023_812_MOESM2_ESM.pdf]

Tabelle 1

resorbierbare Nahtmaterialien		EASSI Symbole	EASSI Symbole	Abkürzung Rohstoff	Handelsname Medtronic®	Covidien Tyco (USSC/	Ethicon ®	B. Braun ®	Resorba®	Serag-Wiessner®	ungefärbt	Farbe	Fadenaufbau	Beschichtung	Widerhaken	Reissfestigkeit I	Reissfestigkeit II	Reisskraft ca. [T] 50%	Reisskraft ca. [T] 0%	Resorption komplett [T]	Hinweis
**Polyglykolsäure**				PGA	Marlin?			Safil					geflochten	+				14-16	28-35	90	
**Polyglykolsäure**				PGA	Marlin rapid?								geflochten	+				7	21	42	
**Polyglykolsäure**	Polyglyton 621			PGA		Caprosyn	Vicryl rapid	Safil Quick			+		geflochten	Magnesium Stearat				5 6-8		42 45-50	
**Polyglykolsäure**				PGA		Polysorb/ Dexon	Vicryl	Safil		Serafit	+	Violett	geflochten	Polyglykonat?				18-21		60-90	
	lactomer 9–1					Polysorb															
**Polyglykolsäure**				PGA						Serafit	+	Violett	geflochten	+				15.20		60-90	
**Polyglykolsäure**				PGA					PGA resoquick ™	Serapid	+		geflochten	+				5-7		42	
**Polycaprolacton-Lactid (PCL)**				PCL									monofil	+				42-50	90-100	180-210	
**Polyglykolsäure-Caprolacton**	Poly(Glykolsäure-co-ε-Caprolacton)								Glycolon	Serafast	+	Violett	monofil	⌀				8-13		90-120	
				PGA-PCL				Quill Monoderm						⌀	+	7 T 50-62%	14 T 27-51%				
**Polyglactin 910**				PGA	Velosorb Fast	Maxon	Vicryl rapide	Novosyn Quick					geflochten	Polyglactin 370 +Calciumstear at				5	12	42	
**Polyglactin 910**	Copolymer Glykolit (Polyglactin 370)+ 10% L-Laktat (B)			PGLA90	Polysorb		Vicryl	Novosyn		Serafit			geflochten					21	35	56-70	
**Polyglactin 910**							Vicryl Plus						geflochten					2135		56-70	
**Poly-p-dioxanon**					Marisorb								monofil					42-50	90-100	180-210	
**Glykolid–ε- Caprolacton-Mischpolymerisat**					Macricryl								monofil					7	28-35	90-120	
**Polyglyton621**	Glykonat			PGC 25	Biosyn	Biosyn	Monocryl	Monosyn		Serafast	B +	Violett	monofil	⌀			14 T 50%	7	28 12-15	90-120 B60-90	
	Glykonat				Caprosyn		Monocryl	Monosyn Quick			+		monofil	⌀		5 T 70-80%	10 T 20-30%			56	
	antibakteriell beschichtet						Monocryl +														
**Polyglecaprone 25**	Poly-Dioxane??			PGC 25		Maxon	PDS II Monocryl Plus					Violett	monofil	⌀				7	28-35	90-120 180-210	
**Poly-p-dioxanon**	Poly-p-dioxanon			PDX	Maxon		PDSII	MonoPlus		Serasynth			monofil	⌀			28 T 50-70%			180-220	
**Polydioxanon**				PDO			PDS						monofil					60% 42T	70	180-240	
**Polydioxanon**				PDO						Serasynth		Violett	monofil	⌀				28-42		180-210	
**Polydioxanon**				PDO						Serasynth Loc		Violett	monofil	⌀				28-42		180-210	
**Polydioxanon**				PDO				Quill PDO					monofil		+	USP ≥3/0: 2 - 4 Wochen 80 % 6 Wochen 40-70 % USP 4/0: 2 Wochen 67 % 4 Wochen 50 % 6 Wochen 37 %				120-180	
**Polydioxanon**	Poly(P-Diaxanon)			PDO					PDO Resorba		+	Violett	monofil					42		175-217	

resorbierbare Nahtmaterialien		EASSI Symbole	EASSI Symbole	Abkürzung Rohstoff	Handelsname Medtronic®	Covidien Tyco (USSC/	Ethicon ®	B. Braun ®	Resorba®	Serag-Wiessner®	ungefärbt	Farbe	Fadenaufbau	Beschichtung	Widerhaken	Reissfestigkeit I	Reissfestigkeit II	Reisskraft ca. [T] 50%	Reisskraft ca. [T] 0%	Resorption komplett [T]	Hinweis
	antibakteriell beschichtet						PDS Plus														
Polydioxanone				PDO			PDS Kordel						geflochten								
Glycomer 631	glycolide, dioxanone and trimethylene carbonate					Biosyn															
Poly-4-Hydroxybutyrat								2-0 - 1					monofil	ø		USP 2/0, 3/0: 1. Monat<br>3 Monate<br>5 Monate<br>USP 1, 0:					
Catgut	Collagen treated with salt					chromic gut	chromic gut	Softcat Chrom			+		verdrillt	ø				14-21		90	
Catgut						gut	gut	Softcat plain			+		verdrillt	ø				7-10		70	
nicht resorbierbare Nahtmaterialien																					
Polyvinylidenfluorid				PVDF	Marilon								monofil	ø							
Polyamid				PA						Nylon		Schwarz	monofil	ø							
Polyamid				PA						Seralon	+	Blau	monofil	ø							
Polyamid	Polyamid titanisiert			PA/Ti						Seratan	+	Blau	monofil	+							
Polyamid	Nylon			PA		Dermalon	Ethilon	Dafilon				Blau	monofil	ø							
Polyamid	Nylon			PA			Nuralon	Trelon			+	Schwarz	pseudomonofil	Silikon							
Polyamid				PA						Supramid	+	Schwarz	gezwirnt	ummantelt							
Polyamid 6/6.6	(Copolymerisat) Fadenseele Polyamid 6.6 (Polymer von Hexamethylendiamin und			PA					Supramid (Sw W)		+	Schwarz Weiss	monofil	ø							allmähliche Degeneration, Hautnähte
Polyamid 6/6.6	(Copolymerisat) Fadenseele Polyamid 6.6 (Polymer von Hexamethylendiamin und			PA				Supramid	Supramid (Sw W)		+	Schwarz	pseudomonofil	ø							allmähliche Degeneration, Hautnähte
Polyamid 6/6.6				PA				Dafilon	Nylon		+ PA6	Blau PA6/6.6 Schwarz	monofil								
Polyamid 6/6.6	spezielle Bearbeitung: weich geschmeidig								Resolon			Blau	monofil								
Polyester				PET		ø	Mersilene	Dagrofil				B Grün + weiß	geflochten	ø							
Polyester				PET						Sulene	+	Grün	multifil	+							
Polyester	Polykondensation Ethylenglykol + Terephthalsäure			PET		Ticron / Surgidac	Ethibond	Premicron/ Synthofil	Polyester	Terylene	+ (SW)	Weiß&Grün (E) grün	geflochten	Silikon / PVA							
Polypropylen				PP	Maprolen				Mopylen				monofil								
Polypropylen				PP			STRATAFIX Spiral Polypropylen														
Polypropylen				PP				Quill Polypropylen					monofil		+						
Polypropylen				PP			Prolene						monofil								
Polypropylen, Polyethylen				PP	Suripro II Surgipro	Surgipro Novafil	Prolene	Optilene				Blau	monofil	ø							

resorbierbare Nahtmaterialien		EASSI Symbole	EASSI Symbole	Abkürzung Rohstoff	Handelsname Medtronic®	Covidien Tyco (USSC/	Ethicon ®	B. Braun ®	Resorba®	Serag-Wiessner®	ungefärbt	Farbe	Fadenaufbau	Beschichtung	Widerhaken	Reissfestigkeit I	Reissfestigkeit II	Reisskraft ca. [T] 50%	Reisskraft ca. [T] 0%	Resorption komplett [T]	Hinweis
**Polyethylenterephthalat**			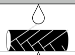	PET	Polyester								geflochten	+							
**Polyethylenterephthalat**			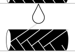	PET	Polyester								geflochten								
**Poly (Hexafluoropropylen-VDF)**							PRONOVO														
**Naturseide**			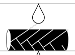	SILK	Naturseide-S					Seraflex			geflochten	+							
**Naturseide**			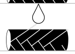	SILK		Sofsilk	Perma-Hand-Seide	Silikam				BSchwarz/BWeiß/Blau	geflochten,	Wachs, Silikon							
**Stahldraht**		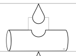		STEEL	Stahldraht					Seranox			monofil								
**Stahldraht**		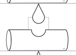		STEEL	Stahldraht								geflochten								
**Stahldraht**		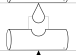		STEEL	Steel		Stainless steel	Steelex					monofil verdrillt	B Polyethylenhülle							
**Polyvinylidenfluorid**		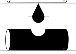		PVDF								Blau	monofil								
**Ligaturen/Unterbindungen**																					
**Polyglactin 910**							Vicryl						geflochten								
**Polyglecaprone 25**							MONOCRYL						monofil								
**Polydioxanone**				PGACL			PDS						monofil								
**Polyester**				PET			Ethibond						geflochten								
**Polyester**				PET			Mersilene						geflochten								
**Seide**							Perma-Hand-Seide						geflochten								
